# Autonomic dysfunction in women with polycystic ovary syndrome

**Published:** 2015-01

**Authors:** Zainab Hasan Hashim, Farqad Bader Hamdan, Anam Rashid Al-Salihi

**Affiliations:** 1 *Department of Physiology, College of Medicine, Al-Nahrain University, Baghdad, Iraq.*; 2 *The High Institute of Infertility Diagnosis and Assisted Reproductive Technologies, Al-Nahrain University, Baghdad, Iraq.*

**Keywords:** *Polycystic ovarian syndrome*, *Sympathetic skin response*, *Heart rate variability*, *Valsalva ratio*

## Abstract

**Background:**

Central obesity and hyperinsulinaemia of polycystic ovary syndrome (PCOS) are associated with chronic sympathetic over activity.

**Objective:**

To evaluate the autonomic functions and to indicate the superiority, if any, for those functions in the diagnosis of sympathetic over activity in PCOS women.

**Materials and Methods:**

Sixty-four PCOS patients and 40 women served as the control group were studied. The two groups were subdivided according to the body mass index (BMI) into two obese and non-obese groups. Waist:hip ratio (WHR), plasma epinephrine level was estimated, sympathetic skin response (SSR); postural orthostatic tachycardia syndrome, heart rate variability (HRV), and valsalva ratio were measured in both groups.

**Results:**

Compared to the control group, obese PCOS patients demonstrated higher BMI and WHR, reduced palmar SSR latency and higher amplitude, altered HRV, higher plasma epinephrine level, and rapid pulse rate. Moreover, non-obese patients show reduced palmar SSR latency and higher amplitude, higher plasma epinephrine level, and higher pulse rate. BMI and WHR of the patients were positively correlated with plasma epinephrine level; while the HRV was negatively correlated WHR.

**Conclusion:**

Women with PCOS exhibits altered autonomic function and sympathoexcitation is more pronounced in obese than non-obese patients; therefor the SSR could be useful auxiliary electrophysiological test to predict autonomic dysfunction in those patients. Receiver operating characteristics curve demonstrate the pulse rate in standing position as the autonomic function test that is superior to others in predicting sympathetic over activity in those patients.

## Introduction

Although the clinical manifestations of polycystic ovary syndrome (PCOS) are heterogeneous, the hallmarks of the syndrome remain anovulation, androgen excess, central obesity, and insulin resistance. Many of these features are associated with chronic sympathetic dominance (1-3). It has therefore been suggested to account for at least a part of the syndromes etiology (3, 4). Heart rate variability (HRV) was the conventionally accepted term to describe variations of both instantaneous heart rate (HR) and R-R intervals (5). Although patients with PCOS have pronounced metabolic abnormalities, a few studies have shown slight impairment in HRV in those women (6-8). The Valsalva ratio (VR) is the maximum HR during the Valsalva maneuver divided by the slowest HR after the Valsalva maneuver. It serves as a more general cardiovascular autonomic nervous system (ANS) test, incorporating cardio vagal (parasympathetic) and sympathetic adrenergic function activity as well as vascular sympathetic NS activity (9). Postural orthostatic tachycardia syndrome (POTS) is characterized by orthostatic tachycardia without significant hypotension (10). 

The basal HR which is mainly under vagal modulation is reported to be high in women with PCOS as a result of decreased vagal activity (11). Moreover, researchers observed raised systolic and diastolic blood pressure (BP) in those women and attribute this increment to increased sympathetic drive as regulation of BP is mainly under sympathetic control (12). Sympathetic skin response (SSR) is a potential change recorded from the skin and dictates sympathetic sudomotor nerve fibers activity. It is a result of polysynaptic reflex arch activation. The effectors of the reflex arch and most probably generators of potential change are activated eccrine sweat glands with cholinergic mediation via postganglionic non-myelinated (type C) fibers (13). 

To our best knowledge, no comparable data in the literatures study SSR in women with PCOS. The study was conducted aiming to evaluate autonomic functions in women with PCOS and to indicate the superiority, if any, of these functions in the diagnosis of sympathetic overstimulation in women with PCOS.

## Materials and methods

This is a cross-sectional study of 64 women with PCOS attends the High Institute for Infertility Diagnosis and Assisted Reproductive Technologies, Baghdad, Iraq for the period from March 2012 to March 2013. They were divided into two subgroups; group Ia comprised 32 obese PCOS patients with BMI ≥30 kg/m^2^ with age range between 18 and 36 years (mean±SD: 27.15±4.50 yr) and group Ib which include 32 non-obese PCOS patients with BMI ≤30 kg/m^2^ and age range between 19 and 33 years (mean±SD: 25.29±3.47 yr). 

Forty healthy women free from PCOS comprises the control group were also studied and divided into two groups; group IIa which consist of 20 obese subjects with BMI ≥30 kg/m^2^ with an age range between 21 and 38 years and group IIb that encompassed another 20 non-obese subjects with BMI ≤30 kg/m^2^ with an age range between 17 and 36 years. The patients were diagnosed with PCOS according to the Rotterdam guidelines (at least two of the following criteria: 1. oligo-anovulation, 2. polycystic ovaries determined by ultrasound, 3. clinical and/or biochemical signs of hyperandrogenism). 

Those patients with thyroid dysfunction, hyperprolactinaemia, and congenital adrenal hyperplasia were excluded from this study. All the patients and control subjects were subjected to full medical and gynecological history and complete physical examination concentrating on hirsutism, alopecia and acne. The weight, height, and waist circumference (measured at the midpoint between the lower rib margin and the iliac crest) (14) were recorded. BMI was then calculated (current weight in kilograms divided by square of height in meters). Three ml of blood following 30 min of rest in supine position and another 3 ml after 10 min of standing were taken to measure epinephrine level.

The studied patients and control subjects were subjected to 15 min Holter monitoring (3-channel recorders Schiller MT 101, Swiss). Holter electrocardiography traces were analyzed automatically and any artifacts, pauses and conduction disturbances that might happen accidentally during recording were eliminated manually by the operator then after. HRV was analyzed in the time domain method in accordance with standards and the following parameters were analyzed; the standard deviation of the normal-to-normal interval (SDNN) measured in millisecond (msec), the standard deviation of the average of NN intervals (SDANN) measured in msec, the square root of the mean of the sum of squares of differences of successive NN intervals (rMSSD) measured in msec, and the number of pairs of adjacent NN intervals differing by more than 50 msec in the entire recording divided by the total number of all NN intervals (PNN50). 

During the last 10-15 seconds of Holter recording, VR estimated while the subject in lying position. The individuals were asked to do Valsalva maneuver by exhaling air and closing nose and mouth, maintaining an expiratory pressure 40 mm Hg with mouth piece. Then the ratio was calculated by dividing the longest R-R interval after maneuver to the shortest R-R interval during or shortly after maneuver. Normal ratio was estimated to be >1.20 as normal and a ratio of ≤1.20 regarded as abnormal. An increase in the HR from supine to upright position for more than 30 beats per minutes or HR increase more than 120 beats per minutes within 10 min of head up tilt will denotes the symptoms of orthostatic intolerance. BP also is measured in the supine followed by standing position. This test was done with Bestmed machine (Multi-parameters patient monitor). 

The SSR was provoked using an electric single square pulse (0.5 msec intensity adjusted to 20-30% above the motor threshold) to stimulate the median nerve or the tibial nerve according to standard method using micromed 4-channel electromyograph machine, Italy (15). The amplitudes and latencies of the SSR were recorded and the response with the highest amplitude measured from peak to peak was chosen for the analysis. 

**Ethical considerations**

The Institute Review Board of the College of Medicine, Al-Nahrain University approved the working protocol, and the written consents of the informed patients and controls were received and then the research was started. 

**Statistical analysis**

Statistical analysis were performed Statistical Package for the Social Sciences, version 16.0, SPSS Inc, Chicago, Illinois, USA. Unpaired two-tailed student’s *t* tests were used to determine differences between groups, and Pearson correlation was calculated for the correlation between two quantitative variables with its *t* test for testing the significance of correlation. The correlation coefficient value r either positive (direct correlation) or negative (inverse correlation). Receiver Operating Characteristics curve (ROC curve) analysis was performed to know the sensitivity and specificity for each autonomic function test. Significance was defined as a p<0.05. 

## Results

The age of the two groups were not different (25.53±4.78 yr for the control group versus 24.53±4.07 yr of the PCOS patient group). The BMI and WHR were significantly higher in the PCOS patients compared to the control group 36.63±4.23 kg/m^2^ vs. 34.14±3.39 kg/m^2^ (p=0.041) and 0.88±0.05 compared to 0.79±0.11 (p=0.001), respectively. 

Significantly reduced latency and higher amplitude of palmar SSR (p<0.001, p=0.031, respectively) was observed in obese PCOS when compared to obese control women. Furthermore, SDNN and pNN50% parameters of HRV were significantly reduced (p=0.023, p=0.002) in the former group as compared to the latter group. The SSR recorded from the sole and VR were not different between the two groups. The pulse rate in supine and standing positions was significantly higher (p=0.003, p<0.001, respectively) in the obese PCOS patients when compared to the obese control women. 

BP in standing and supine positions was not different between the two groups. Epinephrine level was significantly higher in obese PCOS patients as compared to obese control group (p˂0.001) (Table I). Plasma epinephrine level at lying but not standing position was higher (p˂0.001) in the non-obese PCOS when compared to the non-obese control group. Palmar SSR latency was significantly reduced and the amplitude was significantly higher (p=0.043, p*=*0.002, respectively) in non-obese PCOS when compared to non-obese control women, whereas HRV, VR and plantar SSR were not different between the two groups. The pulse rate in supine and standing position was significantly higher (p=0.005; p˂0.001), respectively in non-obese PCOS patients as compared to the control group (Table II).

**Figure 1:**
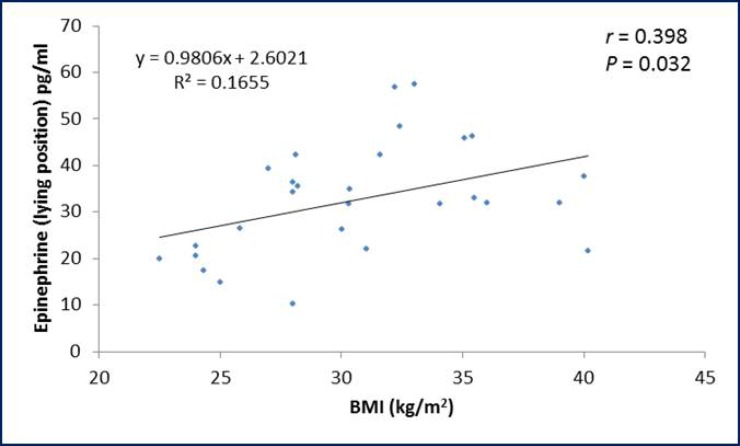
Correlation of BMI and plasma epinephrine level in lying position in PCOS patients

**Figure 2:**
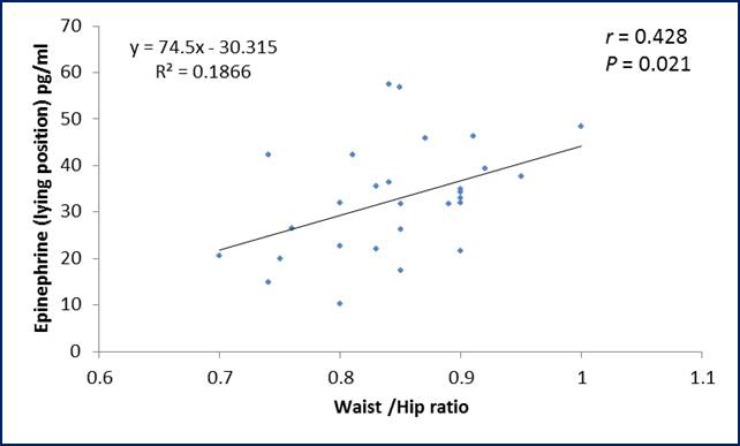
Correlation of waist/hip ratio with plasma epinephrine level in lying position in PCOS patients.

**Figure 3:**
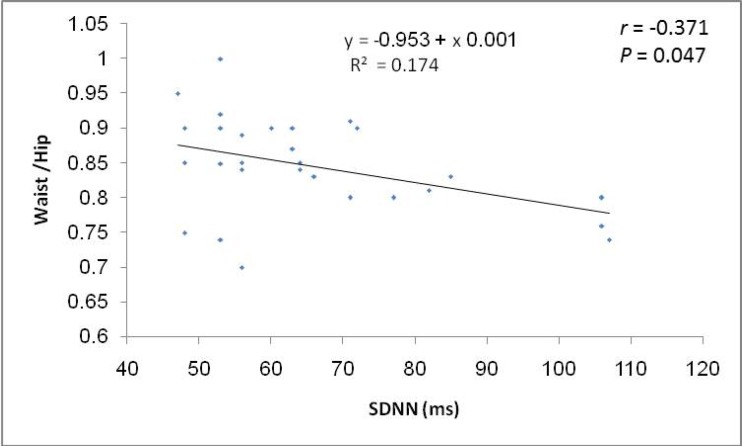
Correlation between waist: hip ratio and SDNN of heart rate variability in PCOS patients.

The ROC curve showed that pulse rate in standing position has the highest area under the curve followed in sequence by palmar SSR latency, pulse rate in supine position, palmar SSR amplitude, pNN50% and systolic BP in supine position (Table III). Considering the specificity and sensitivity of the autonomic function that showed significant difference between PCOS patients and control group; ROC curve demonstrate that the pulse rate in standing position show the highest specificity (94.4%) and sensitivity (81%) as compared to the other tests (Table IV). The BMI and WHR of PCOS patients were positively correlated with plasma epinephrine level in lying position (Figures 1, 2). Moreover, a negative correlation (r=-0.371; p=0.047) was observed between SDNN parameter of the HRV and WHR in PCOS patients (Figure 3).

**Table I:** Autonomic functions of the obese PCOS patients versus obese control women

**Autonomic function**		**Obese control women (n=20)**	**Obese PCOS patients (n=32)**	**p-value**
Palmar SSR				
	Latency (sec)	1.53 ± 0.14	1.29 ± 0.18	<0.001
	Amplitude (mV)	1.08 ± 0.96	1.97 ± 1.13	0.031
	Duration (sec)	4.2 ± 0.7	4.3 ± 1.13	0.78
Plantar SSR				
	Latency (sec)	1.89 ± 0.4	2.05 ± 0.24	0.215
	Amplitude (mV)	0.74 ± 0.6	0.57 ± 0.46	0.344
	Duration (sec)	3.13 ± 0.6	3.56 ± 0.87	0.148
HRV				
	SDNN	83.25 ± 27.47	63.56 ± 16.22	0.023
	SDANN	54.0 ± 28.86	38.5 ± 18.65	0.075
	rMSSD	31.25 ± 22.6	26.31 ± 10.71	0.46
	pNN50%	12.24 ± 3.17	5.42 ± 4.63	0.002
Valsalva ratio		1.25 ± 0.04	1.2 ± 0.15	0.313
Pulse rate/min				
	Supine	72.5 ± 5.84	82.88 ± 9.83	0.003
	Standing	83.25 ± 9.44	104.75 ± 8.84	˂0.001
Systolic BP (mmHg)				
	Supine	130.5 ± 7.59	133.94 ± 12.67	0.494
	Standing	132.0 ± 8.58	129.69 ± 12.91	0.565
Diastolic BP (mmHg)				
	Supine	79.25 ± 8.62	78.63 ± 10.83	0.858
	Standing	87.75 ± 10.69	85.25 ± 8.75	0.439
Plasma epinephrine (pg/ml)				
	Lying	6.87 ± 2.28	28.74 ± 17.43	˂0.001
	Standing	11.07 ± 5.63	37.33 ± 11.05	˂0.001

Data are presented as mean±SD.

Unpaired two-tailed t- test was used

SSR: sympathetic skin response

HRV: heart rate variability

BP: blood pressure

SDNN: standard deviation of the normal-to-normal interval

SDANN: standard deviation of the average of NN intervals

rMSSD: square root of the mean of the sum of squares of differences of successive NN intervals

PNN50: number of pairs of adjacent NN intervals differing by more than 50 msec in the entire recording divided by the total number of all NN intervals

**Table II:** Autonomic functions of the non-obese PCOS versus non-obese control women

**Autonomic Function**			**Non-obese control women (n=20)**	**Non-obese PCOS patients (n=32)**	**p-value**
Palmar SSR					
	Latency (sec)		1.5 ± 0.11	1.38 ± 0.17	0.043
	Amplitude (mV)		0.85 ± 0.66	2.15 ± 1.44	0.002
	Duration (sec)		4.53 ± 0.97	4.08 ± 0.56	0.182
Plantar SSR					
	Latency (sec)		2.03 ± 0.27	1.88 ± 0.36	0.24
	Amplitude (mV)		0.49 ± 0.23	0.72 ± 0.54	0.194
	Duration (sec)		3.86 ± 0.66	4.0 ± 0.918	0.616
HRV					
	SDNN		70.67 ± 24.06	68.33 ± 19.63	0.778
	SDANN		52.17 ± 20.87	41.5 ± 21.89	0.206
	rMSSD		28.0 ± 19.81	30.33 ± 14.54	0.73
	pNN50%		10.32 ± 2.03	8.4 ± 5.56	0.965
Valsalva ratio			1.28 ± 0.1	1.25 ± 0.14	0.596
Pulse rate/min					
		Supine	73.83 ± 7.69	83.42 ± 10.77	0.005
		Standing	89.78 ± 5.58	106.25 ± 13.46	˂0.001
Systolic BP (mmHg)					
		Supine	110.33 ± 16.74	125.58 ± 11.3	0.003
		Standing	121.5 ± 8.19	126.67 ± 11.55	0.191
Diastolic BP (mmHg)					
		Supine	74.89 ± 8.21	73.08 ± 8.17	0.596
		Standing	79.5 ± 7.29	83.17 ± 6.78	0.247
Plasma epinephrine (pg/ml)					
		Lying	6.86 ± 2.27	24.3 ± 12.48†	˂0.001
		Standing	15.8 ± 6.75	26.51 ± 10.57	0.057

Data are presented as mean±SD.

Unpaired two-tailed t- test was used

BMI: body max index

SSR: sympathetic skin response

HRV: heart rate variability

BP: blood pressure

SDNN: standard deviation of the normal-to-normal interval

SDANN: standard deviation of the average of NN intervals

rMSSD: square root of the mean of the sum of squares of differences of successive NN intervals

PNN50: number of pairs of adjacent NN intervals differing by more than 50 msec in the entire recording divided by the total number of all NN intervals

**Table III:** Area under the curve of the receiver operating characteristics

**Parameter**	**Area**	**Std. error**	**p-value**	**Asymptotic 95% CI**	
				**Lower bound**	**Upper bound**
Pulse rate (standing)	0.906	0.032	˂0.001	0.844	0.968
SSR latency (msec)	0.797	0.045	˂0.001	0.709	0.884
Pulse rate (supine)	0.773	0.047	˂0.001	0.681	0.865
SSR amplitude (µV)	0.736	0.054	˂0.001	0.631	0.842
pNN 50%	0.719	0.051	˂0.001	0.618	0.82
Systolic BP supine (mmHg)	0.638	0.06	0.025	0.522	0.755

SSR: sympathetic skin response

BP: blood pressure

CI: Confidence interval

PNN50: number of pairs of adjacent NN intervals differing by more than 50 msec in the entire recording divided by the total number of all NN intervals

**Table IV:** Specificity and sensitivity of different autonomic function tests

**Autonomic function tests**	**Specificity (%)**	**Sensitivity (%)**
Pulse rate (Standing position)	94.4	81
Blood pressure mmHg (Supine position)	86.1	36.2
Palmar SSR amplitude (µV)	75	74.1
Palmar SSR Latency (msec)	72.4	52.8
Pulse rate (supine position)	72.2	60.3
pNN50%	63.8	75

SSR: sympathetic skin response

PNN50: number of pairs of adjacent NN intervals differing by more than 50 msec in the entire recording divided by the total number of all NN intervals

## Discussion

We demonstrated high plasma epinephrine level during lying and standing positions in PCOS patients. This could be of obesogenic origin as we noticed a positive correlation between plasma epinephrine level and both of BMI and WHR. PCOS patients of this study exhibited central abdominal obesity and the mechanisms by which central obesity drive an increase in sympathetic activity are not entirely clear. Yet, the fat cells have increased sensitivity to lipolytic agents and/or the factors inducing fat mobilization are turned on (16). This was further supported that adipocytes isolated from the visceral fat depot of women with PCOS had increased catecholamine-stimulated lipolysis (17). 

Of note, sympathetic nerve activity is much more closely associated with visceral than total or subcutaneous fat mass (3). Indeed, sympathetic nerve output is increased in central obesity even in the absence of hypertension (18). The pulse rate was increased in standing position compared to supine position in women with PCOS of the current study without significant changes in upright BP. This finding was in harmony with other studies in which patients with POTS demonstrate a HR increase of ≥30 beat/minute with prolonged standing of 5-30 min (19, 20). This increment could be due to cardiac sympathetic stimulation that mainly reflects augmented compensatory activation in response to excessively decreased venous return to the heart (21, 22). 

Among other mechanisms are the vagal impairment and hyperadrenergic activity supported by the high levels of upright plasma epinephrine (reflecting SNS activation) in this study and marked impairment of cardiac baroreflex sensitivity (23-25). Furthermore, compensatory increase in cerebral sympathetic outflow leads to elevated norepinephrine levels, which result in an increase in HR and myocardial contractility (19, 26). Women with PCOS have a higher level of sympathetic nerve activity (as detected through lower SDNN and pNN50 measurements) than the women in the control group. This finding harmonizes the results of others, which would reflect sympatho-vagal imbalance in patients with PCOS reflected as diminished vagal and an increased sympathetic modulation of sinus node (27-31). 

In the present study, no significant change in the VR was demonstrated in PCOS patients as compared to normal groups. This is because VR serve as a general quantitative cardiovascular ANS test useful for long-term evaluation of severe rather than early detection of mild autonomic dysfunction or a specific parasympathetic or sympathetic NS evaluator (32, 33). Despite their BMI, women with PCOS of our study verified significantly faster latency and enhanced amplitude of the palmar SSR when compared to the control women. Overactivity of the sympathetic system, as seen in PCOS, would be expected to alter skin resistance through its effect on sudomotor fibers, thereby affecting the amplitude of SSR. This would suggest enhanced excitability of the postganglionic sympathetic cholinergic fibers to sweat glands. 

Bidzinska-Speichert stated that PCOS must be suspected in every adolescent girl with menstrual irregularity, hirsutism, obesity, persistent acne vulgaris, scalp hair loss and hyperhidrosis (34). Moreover, Kim and Rosenfield stated that among cutaneous signs of androgen excess is acne, seborrhea, alopecia, or hyperhidrosis (35). This would stresses that sympathetic over activity did not spares the skin in PCOS patients and emphasize on a link between PCOS, hyperandrogenemia and sympathetic autonomic cholinergic over activity and the latter finding could be involved in the pathogenesis of patients with PCOS. A significant positive correlation was noticed between plasma epinephrine in lying position and either of BMI and WHR, a finding that was also reported by others (36). 

Obesity was found to be a major determinant of sympathetic discharge in women (37). Obese normotensive women had a greater basal muscle sympathetic nervous activity than lean women (38). de Sá *et al* found that there was significant negative correlation between BMI and SDNN indicating a decrease in the autonomic modulation of HR with increasing weight (2). This finding is compatible with the present study, in that there is significant inverse correlation between WHR and SDNN in PCOS patients. From ROC curve, the pulse rate in standing position was the autonomic function test that is superior to others in predicting sympathetic over activity in PCOS patients. The current evidence would support the view that plasma epinephrine concentration was increased in study group than in normal population, which suggests a chronic elevation of sympathetic activity in PCOS. This indicate that they have decreased dynamic activity in their autonomic function, possibly by decreased activity in the parasympathetic component and increased activity in the sympathetic component. This sympathovagal imbalance might expose them to cardiovascular morbidities. 

**Limitation**

No specific limitation was present in this study, yet the tests require a long time and needs patience.

## Note

***This article extracted from Ph.D. thesis. (Zainab Hasan Hashim)***
